# Characterization and Safety Assessment of a Novel Antioxidant Excipient from Sustainable Recovery of Grape Processing Waste Bentonite Designed to Develop a Thermosensitive Buccal Spray for Oral Cavity Wellness

**DOI:** 10.3390/pharmaceutics16121612

**Published:** 2024-12-19

**Authors:** Giulia Di Prima, Elena Belfiore, Cecilia La Mantia, Serena Indelicato, Giuseppe Avellone, Viviana De Caro

**Affiliations:** 1 Dipartimento di Scienze e Tecnologie Biologiche, Chimiche e Farmaceutiche (STEBICEF), University of Palermo, Via Archirafi 32, 90123 Palermo, Italy; cecilialamantina000@gmail.com (C.L.M.); serena.indelicato@unipa.it (S.I.); beppe.avellone@unipa.it (G.A.); viviana.decaro@unipa.it (V.D.C.); 2 Dipartimento di Medicina di Precisione in Area Medica, Chirurgica e Critica (Me.Pre.C.C.), University of Palermo, Via L. Giuffrè 5, 90127 Palermo, Italy; elena.belfiore@unipa.it; 3 Centro Interdipartimentale di Ricerca Riutilizzo Bio-Based Degli Scarti da Matrici Agroalimentari (RIVIVE), University of Palermo, 90128 Palermo, Italy

**Keywords:** cosmeceutical, polyphenols, bentonite, waste recovery, safety assay, PEG200, propylene glycol, in situ gelling, oromucosal gel

## Abstract

Background/Objectives: Nowadays, sustainability efforts focus on extracting natural cosmeceutical ingredients, such as polyphenols, from agri-food waste, for example, black bentonite (BB). The aims of this work were to validate an antioxidant cosmetic ingredient obtained from the waste BB and embed it into an ad hoc designed oromucosal spray intended for oral cavity wellness. Methods: Focusing on sustainability, the study tested PEG200, propylene glycol, and their mixtures as unconventional and green extraction solvents, aligned with a waste-to-market approach. The extracts obtained by maceration were characterized through HPLC-DAD and HPLC-MS analyses, DPPH, Bradford, and Folin–Ciocalteu assays. The best P extract was further subjected to OECD-compliant in vitro validation as novel cosmetic raw material and used to prepare a thermosensitive buccal spray for oral daily care. Results: PEG200 enabled the obtainment of a cost-effective polyphenol-rich extract, which was validated as a safe, high value-added cosmetic secondary raw material. The extract was incorporated into a liquid thermosensitive buccal formulation, able to gel once at body temperature and enhance polyphenol accumulation into the oral mucosae even with short contact times. Conclusions: BB is confirmed as a valuable source of polyphenols, and PEG200 represents an effective extraction solvent leading to a novel functional liquid excipient characterized by an OECD-compliant variegate pool of phenols. The buccal spray then proposed represents a valuable, friendly solution for daily oral care, as it is simple to use, as well as the in vitro and ex vivo tests carried out suggested its effectiveness.

## 1. Introduction

The term ‘cosmeceuticals’ was introduced in the 1980s to mention products or ingredients that are borderline between cosmetics and pharmaceuticals [1]. It is a controversial word because, even if it has no meaning under the law in both Europe and the USA, it is quite popular among the scientific community. In the recent literature, several scientists report about cosmeceuticals in terms of topically applied formulations or raw materials containing biologically active ingredients purporting to have medical properties or drug-like benefits [2]. Among them, antioxidants are a relevant class of biocompounds of cosmeceutical interest [3]. Indeed, oxidative stress is the key factor in both physiological processes of cosmetic interest (e.g., aging, photoaging) and pathological mechanisms affecting the skin and the mucosae (e.g., oral lichen planus, oral mucositis). These naturally occurring events or altered conditions are both characterized by an imbalance between the oxidative species (e.g., ROS, RNS, etc.) and the physiological antioxidant defense (e.g., glutathione, SOD, etc.) [4,5,6]. Among the most studied and used antioxidants, polyphenols have certainly been under the spotlight for years due to their wide range of biological properties, also including anti-inflammatory, cardioprotective, chemopreventive, anticancer, and neuroprotective actions, which strongly justify their possible classification as cosmeceutical active ingredients [7,8,9,10,11]. Polyphenols are naturally occurring molecules widely produced by plants and thus found in a variety of fruits [12]. Due to their wide range of biological actions, recently, numerous studies have focused on their recovery and extraction from several biological sources [13,14,15,16]. Polyphenol intake as nutraceuticals could be useful to promote general human health; however, mainly due to their poor water solubility, instability (due to light exposure, alkaline pH, and temperature higher than 40 °C [17]), and high susceptibility to first-pass effect metabolism, their bioavailability is extremely low, and consequently, to deal with skin or mucosal disorders, it should be more effective to administer them locally [18]. To achieve this aim, polyphenols could be recovered from several natural sources, as previously mentioned. Furthermore, to accomplish the growing demand for actives from a sustainability perspective, scientists are recently focusing on wastes to be recycled and valorized to recover active molecules such as polyphenols. In this context, the primary investigation focuses on waste materials derived from the agri-food sector [19,20,21]. Among the agri-food by-products, the organic ones are basically the most considered. As an example, grapes are naturally rich in polyphenols [22], and thus the wastes and by-products coming from the winemaking and grape processing industries have been recently valorized as an innovative source of polyphenols. However, this circular economy approach has generally been performed by selecting the organic by-products, such as peel, pomace, and seeds [23,24,25], while the grape processing sector, as well as other supply chains, also produce huge amounts of inorganic waste, which could be a relevant source of bioactive molecules. A valuable example is represented by the black bentonite (BB) used to fine white grape musts and wines. It is a mineral clay mixed with active carbon, which is used to remove protein haze, thereby leading to more stable and clear final products able to meet consumers expectations. The BB is the most used clarifying agent, and, importantly, it cannot be reconstituted and reused. Thus, an enormous quantity of this waste is produced per year and ultimately destined for disposal [26]. Nevertheless, black bentonite, together with proteins, can also retain certain amounts of the precious polyphenols contained in the white grape must/wine. The latter can be extracted by means of green extraction procedures involving the employment of unconventional extraction solvents chosen, according to a waste-to-market approach, among the well-known liquid hydrophilic excipients used in cosmetics and pharmaceuticals [27]. Based on these considerations, the aims of this work were to validate the reproducibility of polyphenols’ sustainable extraction from the waste BB by selection of the best solvent/mixture, demonstrate the safety of the extract as new raw material/excipient in accordance with the OECD Guidelines for the Testing of Chemicals, and directly employ this extract to formulate a valuable buccal in situ gelling product of cosmeceutical interest due to its antioxidant properties and ability to promote polyphenols’ interaction and accumulation into the oral mucosae. The novelty of the here-discussed research relies on numerous aspects, including the choice of the waste to be valorized, the selection of the unconventional extraction “solvent”, the validation of a novel safe cosmetic raw material, and the formulation of a liquid buccal spray able to gel once at body temperature and to maximize polyphenol accumulation into the buccal and sublingual mucosae, leading to benefitting from their cosmetic action when inserted into a daily oral care routine.

## 2. Materials and Methods

### 2.1. Materials

The waste Black Bentonite (BB; Enobent^®^ Standard + activated carbon 1:1 *w*/*w*) was supplied by Bono & Ditta S.p.A. (Campobello di Mazara, TP, Italy) after its use as a fining agent (100 g of BB to fine 1 hL of must from white biological grapes). Polyvinylpyrrolidone K30 (PVP K30), pluronic F-127, propylene glycol (G), and trans-resveratrol (RSV) were purchased from A.C.E.F. Spa (Fiorenzuola D’Arda, PC, Italy). Polyethyleneglycol 200 (PEG200), bovine serum albumin (BSA), ascorbic acid, potassium sorbate, sodium metabisulfite, urea, sodium dehydrocholate, and 2,2-diphenyl-1-picrylhydrazyl free radical (DPPH) were obtained from Carlo Erba (Milan, Italy). Benzyl alcohol was obtained from Galeno (Carmignano, Italy). Quercetin (QRC) and xylitol were supplied by Farmalabor (Canosa di Puglia, BT, Italy). Gallic Acid (GA), Bradford and Folin–Ciocalteu reagents, sodium lauryl sulfate (SLS), methyl acetate (MA), and 2,4-dinitrochlorobenzene (DNCB) were purchased from Merck (Darmstadt, Germany). Quercetin, gallic acid, resveratrol, apigenin-7-glucoside, apigenin, chlorogenic acid, hydroxycinnamic acid, kaempferol, vanillic acid, ferulic acid, caffeic acid, catechin, epicatechin, rutin, syringic acid, and gentisic acid standards for LC-MS/MS analyses were purchased from Merck (Darmstadt, Germany). The EpiDermTM Model (Batch 36186) and EpiOcular™ EIT Model (Batch 34994) were supplied by MatTek (Bratislava, Slovakia). Phosphate-buffered saline (PBS) and sterile water were purchased from Euroclone S.p.A. (Pero, MI, Italy). Cell culture medium (GIBCO, 31870-02) was supplied by Thermo Fisher Scientific (Waltham, MA, USA). Citrate buffer pH 5.5 was prepared by dissolving 3.024 g of anhydrous sodium citrate and 0.636 g of citric acid monohydrate in 1 L of distilled water. 9 g of NaCl were dissolved in 1 L of distilled water to prepare the isotonic saline solution (0.9% *w*/*v*). While 9 g of NaCl and 50 g of trehalose were dissolved in 1 L of distilled water to prepare the isotonic saline solution containing 5% *w*/*v* of trehalose. The simulated salivary fluid pH 6.8 was prepared according to the literature [28]. All other chemicals and solvents (analytical grade) were obtained from Carlo Erba and were used without further purification. Porcine specimens were kindly supplied by the Azienda Agricola Mulinello S.r.l. (Leonforte, EN, Italy).

### 2.2. Recovery, Storage, and Pretreatments of Waste BB

Immediately after filtration and squeezing, the BB was recovered and kept at −20 °C. Afterwards, about 3 kg of BB, considered as belonging to the same batch, were transferred into refrigerated boxes to the laboratories of the University of Palermo. Prior to storage at −80 °C (Thermo Forma ultra-freezer model 902 Thermo Fisher Scientific, Waltham, MA, USA), the recovered batch of BB was subjected to standardized pre-treatments including pulverization, mixing, sieving, and aliquotation.

### 2.3. Green Extraction of Polyphenols

The extractions were carried out by maceration as previously reported by treating 3 g of BB with 12 g of the extraction solvent/mixture as reported in Table 1 [27,29]. After maceration, centrifugation, and filtration, the recovered extracts were stored in amber glass vials at 4 °C in the dark. Each extraction was repeated six times (n = 6).

### 2.4. Solvent Loss %, Density, and pH After Water Dilution

The solvent loss % after the extraction procedure was calculated as follows:

$$Solvent\;loss\;\%=\frac{starting\;amount\;of\;solvent\;\left(g\right)-amount\;of\;recovered\;extract\;\left(g\right)}{starting\;amount\;of\;solvent\;(g)}.$$

Results are reported as means (n = 6) ± standard error (SE). The density was mathematically evaluated by carefully weighing 500 µL of each extract (Mettler Analytical Balance, Columbus, OH, USA, Mod. AE 240). Results are reported as g/mL. Finally, to evaluate the pH after water dilution, samples were prepared at a fixed concentration (100 mg/mL) and analyzed by a pH meter HI 2211 pH/ORP Meter, Hanna Instrument (Woonsocket, RI, USA). Both the density and the pH measurements were repeated three times for each extract (n = 18), and results are reported as means ± SE.

### 2.5. Quantification of GA, RSV, and QRC by HPLC-DAD Analysis

Samples were analyzed as previously reported [27,29] after proper dilution (1:1 *v*/*v*) with methanol. The chromatographic separation was achieved by using an HPLC Agilent 1260 Infinity Instrument equipped with a G7129C automatic vial sampler (injected volume: 20 µL; column temperature: 25 °C), a Quaternary Pump G1311B, a Diode Array Detector 1260 Infinity II, and a computer-integrating apparatus (OpenLAB CDS ChemStationWorkstation, Stockholm, Sweden). An Ace^®^ Excel Super C18 reversed-phase column (125 mm × 4.60 mm; 100 Å; 5 µm column) was used as the stationary phase while a mixture of 0.1% (*v*/*v*) TFA water solution (solvent A) and acetonitrile (solvent B) was employed as the mobile phase. The chromatographic separation was reached at a 1 mL/min flux in gradient conditions as follows: 0–2 min isocratic conditions A:B = 90:10; gradient from 2 min A:B = 90:10 to 22 min A:B = 5:95; 22–23 min isocratic conditions A:B = 5:95; gradient from 23 min A:B = 5:95 to 25 min A:B = 90:10; and finally, 25–27 min isocratic conditions A:B = 90:10. DAD spectrum covered the range between 190 and 650 nm. In these conditions, the retention times of gallic acid (GA), resveratrol (RSV), and quercetin (QRC) were 2.7, 11.1, and 11.7 min, respectively. For each compound, the appropriate calibration curve was constructed as follows:

- GA: linearity range: 0.1–0.001 mg/mL; λ_max_ = 271 nm; and regression equation: Area = 30,079.84 × [mg/mL] (R = 0.999).
- RSV: linearity range: 0.01–0.0001 mg/mL; λ_max_ = 305 nm; and regression equation: Area = 140,915.24 × [mg/mL] (R = 0.999).
- QRC: linearity range: 0.1–0.001 mg/mL; λ_max_ = 370 nm; and regression equation: Area = 72,464.34 × [mg/mL] (R = 0.999).

Intraday and interday variations were lower than the sensibility. Each experiment was performed in triplicate on each prepared extract (n = 18), and results are expressed as means ± SE.

### 2.6. Determination of the Total Phenolic Content (TPC) by Folin–Ciocalteu Assay

The Folin–Ciocalteu assay was performed as previously reported [27,29]. Specifically, 50 µL of a 100 mg/mL water solution of each extract was added to 2 mL of distilled water, then treated with 130 µL of Folin–Ciocalteu reagent (5 min settle) and subsequently 370 µL of Na_2_CO_3_ solution (0.2 g/mL). After 2 h in the dark, samples were subjected to UV–Vis analysis (Shimadzu UV-1700 PharmaSpec instrument, Kyoto, Japan). Each experiment was performed in triplicate on each extract (n = 18), and results are expressed as mg equivalent of GA per 1 g of extract (means ± SE). To construct the standard GA calibration curve, six standard solutions were prepared and analyzed: linearity range: 0.98–9.80 µg/mL; λ_max_ = 760 nm; and regression equation: Abs = 0.039 + 65.08 × [mg/mL] (R = 0.999).

### 2.7. Evaluation of the Polyphenolic Footprint by HPLC-MS Analysis

The identification of the polyphenols in each extract was performed after appropriate dilution (1:100 *v*/*v*) with methanol, following a procedure adapted from Indelicato et al. [30], which was partially modified to suit the development of an UHPLC/MS-MS method. For the LC-MS/MS experiments, an Ultimate 3000 system connected to a TSQ Quantiva (Thermo Fisher Scientific, San José, CA, USA), a triple quadrupole mass spectrometer, was used. Chromatographic separation was conducted using a Hypersil GOLD C18 reversed-phase analytical column (2.1 mm × 50 mm, 1.9 μm particle size, Thermo Fisher Scientific), maintained at 30 °C, with a 5 μL injection volume. A gradient elution was used, combining mobile phase A, purified water containing 0.1% formic acid (LC-MS grade, Sigma-Aldrich, St. Louis, MO, USA), and mobile phase B, methanol (LC-MS grade, Sigma-Aldrich), at a flow rate of 300 μL/min. The gradient was as follows: 0–2 min, 5% B; 2–10 min, linear increase to 70% B; 10–12 min, linear increase to 100% B; 12–17 min, maintained at 100% B; 17.0–17.1 min, linear decrease to 1% B; 17.1–19 min, maintained at 1% B. The mass spectrometer (QqQ, Thermo Fisher Scientific, Germany) was equipped with a heated electrospray ionization (HESI) source. LC-MS/MS analysis was carried out in negative ion mode, with tuning performed using standard solutions of the analytes at a concentration of 1 ppm in methanol.

The mass spectrometry conditions were as follows: HESI (-), static spray voltage at 2500 V, auxiliary gas pressure at 10 arbitrary units, sheath gas pressure at 50 psi, sweep gas at 1 arbitrary unit, ion transfer tube temperature at 325 °C, vaporizer temperature at 350 °C, with a dwell time of 100 ms; Q1 resolution at 1 Da, Q3 resolution at 0.4 Da, and collision-induced dissociation (CID) gas (Ar) at 2.0 mTorr. Selected reaction monitoring (SRM) was conducted on the deprotonated molecules of each polyphenol ([M-H]-), with SRM transitions detailed in Table S1 (see Supplementary Material). The quantification method involved integrating the areas under all monitored transitions. The analysis was carried out using the following standards: quercetin, gallic acid, resveratrol, apigenin-7-glucoside, apigenin, chlorogenic acid, hydroxycinnamic acid, kaempferol, vanillic acid, ferulic acid, caffeic acid, catechin, epicatechin, rutin, syringic acid, and gentisic acid.

To quantify the phenolic compounds, an external calibration method was used. A methanolic solution containing 5 ppm of each standard was prepared, from which five calibration solutions were created at concentrations of 1 ppm, 500 ppb, 250 ppb, 100 ppb, and 50 ppb for each analyte. The linearity of the calibration curve had a correlation coefficient (R^2^) of 0.99. Data were processed using the Quan/Qual Browser in Trace Finder (Thermo Fisher Scientific, San Jose, CA, USA). Each point on the calibration curve was derived from the average of three independent injections. The limits of detection (LODs) and quantification (LOQs) for each compound in the standard solutions were determined using the blank signal and regression curve (the average of five blanks injected between standards) within the same elution time window as the analytes. LOD was defined as the concentration yielding a signal equivalent to the blank signal plus three times the standard deviation of the blank. LOQ was calculated as the concentration yielding a signal equal to the blank signal plus ten times its standard deviation. The percentage of relative standard deviation (RSD%) for each sample was below 5%. Each experiment was performed in triplicate on each prepared extract (n = 18), and results are expressed as means ± SE.

### 2.8. Evaluation of the Organic Acids Content by HPLC-MS Analysis

The identification of organic acid in each sample was performed after appropriate dilution (1:100 *v*/*v*) with methanol. The LC-MS/MS experiments were conducted using an Ultimate 3000 system connected to a triple quadrupole mass spectrometer, TSQ Quantiva (Thermo Fisher Scientific, San José, CA, USA). The chromatographic column was a Hypersil GOLD C18 reversed-phase analytical column (2.1 mm × 50 mm, 1.9 μm particle size, Thermo Fisher Scientific), with an injection volume of 5 μL. The mobile phases for LC-MS/MS were (A) purified water containing 0.1% formic acid (LC-MS grade, Sigma-Aldrich) and (B) methanol (LC-MS grade, Sigma-Aldrich), and the flow rate was 300 μL/min. Chromatographic separation was achieved using an elution gradient as follows: 0–1.5 min, 2% B; 1.5–2.5 min, linear increase to 25% B; 2.5–3 min, linear increase to 70% B; 3–5 min, maintained at 70% B; and 6–8 min, linear decrease to 2% B. The mass spectrometer (QqQ, Thermo Fisher Scientific, Dreieich, Germany) was equipped with a heated electrospray ionization (HESI) source. LC-MS/MS analysis was carried out in negative ion mode, with tuning performed using standard solutions of the analytes at a concentration of 1 ppm in methanol. The mass spectrometry conditions were as follows: HESI (-), static spray voltage at 2500 V, auxiliary gas pressure at 10 arbitrary units, sheath gas pressure at 50 psi, sweep gas at 1 arbitrary unit, ion transfer tube temperature at 325 °C, vaporizer temperature at 350 °C, with a dwell time of 100 ms; Q1 resolution at 1 Da, Q3 resolution at 0.4 Da, and collision-induced dissociation (CID) gas (Ar) at 2.0 mTorr. Selected reaction monitoring (SRM) was performed on the deprotonated molecules of organic acids ([M-H]-), with SRM transitions detailed in Table S2 (see Supplementary Material). The quantification method involved integrating the areas under all monitored transitions, and MS experiments were carried out using the following standards: lactic acid, tartaric acid, citric acid, and malic acid. In order to quantify organic acids, an external calibration method was used. An aqueous solution containing 100 ppm of each standard was prepared, followed by a working solution at 1 ppm. Five calibration solutions were created at concentrations of 1 ppm, 500 ppb, 250 ppb, and 50 ppb for each analyte. The linearity of the calibration curve had a correlation coefficient (R^2^) of 0.99.

### 2.9. Determination of the Total Protein Content (TPtC) by Bradford Assay

The Bradford assay was performed as previously reported [27,29]. 50 µL of a 100 mg/mL water solution of each extract were added to 600 µL of distilled water, then treated with 200 µL of Bradford reagent. After 30 min in the dark, samples were subjected to UV–Vis measurements. Each experiment was performed in triplicate on each extract (n = 18), and results are expressed as mg equivalent of BSA per 1 g of extract (means ± SE). To construct the standard BSA calibration curve, five standard solutions in ultrapure water were prepared and analyzed: linearity range 2–7 µg/mL; λ_max_ = 595 nm; and regression equation: Abs = 0.1700 + 0.0332 × [µg/mL] (R = 0.998).

### 2.10. Evaluation of the Antioxidant Power by DPPH Assay

The DPPH assay was performed as previously described [29]. Briefly, 2 mL of DPPH stock solution (40 µg/mL in methanol) were inserted into a quartz cuvette and added with 100 µL of a 20 mg/mL methanolic solution of each extract. The solutions were immediately subjected to UV–Vis measurements (Shimadzu UV-1700 PharmaSpec, Kyoto, Japan), which were repeated every 5 min for 1 h. The fresh extraction solvent or mixture was treated analogously to blank control samples. Each experiment was performed in triplicate on each extract (n = 18), and results are expressed as the percentage amount of residual DPPH (means ± SE) as a function of time. To quantify the residual DPPH percentage, the appropriate calibration curve was constructed: linearity range: 4–40 µg/mL; λ_max_ = 515 nm; regression equation: Abs = 0.018 + 28.59 × [mg/mL] (R = 0.999).

Moreover, the antioxidant power of the extracts was reported as mg equivalents of GA (GAE) per 1 g of extract (means ± SE; n = 18). To do this, standard DPPH curves were obtained by analyzing 5 different GA standard solutions, each in triplicate (n = 3). The residual DPPH % values at 10, 30, and 60 min were used to construct 3 calibration curves useful to calculate the antioxidant power of each extract over time. The obtained standard curves are reported in Table 2.

### 2.11. Control Groups

To the aim of detecting any eventual interference from the clarify agent BB, the extraction procedure by maceration with each mixture/solvent was also carried out on fresh (not used) BB. No compounds were recovered from the control BB, and the obtained control samples did not possess any antioxidant power, phenolic and protein contents, nor HPLC-DAD peaks, so results are not reported.

### 2.12. Stability Studies

Six samples of P extract were stored at 4 °C in the dark for a total of 12 months. Monthly, they were subjected again to the previously described HPLC-DAD analysis and DPPH assay to assess their stability at the employed storage conditions. Each stability test was performed in duplicate on each sample (n = 12), and the obtained results are expressed as means ± SE.

### 2.13. In Vitro Skin Irritation Test on Reconstructed Human Epidermis

The in vitro skin irritation test was performed in accordance with [31,32]. The assay was conducted on a 3D human artificial skin model (EpiDermTM) treated with 30 μL of P extract (10% *w*/*v* in sterile water), PBS as a negative control, and SLS (5% *w*/*v* in sterile water) as a positive control for 35 min at 37 °C or 25 min at room temperature. Afterwards, the tissue was washed with PBS and incubated at 37 °C and 5% CO_2_ for 42 h by replacing the culture medium after 24 h. Subsequently, the tissue was incubated with the MTT solution (1 mg/mL) at 37 °C for 3 h. The fluid was then replaced with isopropanol and incubated at room temperature for a further 2 h. Two aliquots of each sample were transferred into a 96-well plate and the absorbance at 570 nm was assessed by a microplate reader (Infinite 200 PRO model, Tecan, Hombrechtikon, Switzerland). The values obtained for the untreated control cells were taken as 100% of cell viability and used as a reference to calculate the cell viability % of each sample. The experiments were performed in triplicate (n = 3), and results are reported as means ± standard deviation (SD).

### 2.14. In Vitro Evaluation of the Eye Irritation Potential

The in vitro eye irritation test was performed in accordance with the OECD 492 guideline for testing of chemicals [33,34,35,36]. The assay was conducted on a 3D human corneal epithelium model (EpiOcular™ EIT) treated with 50 μL of P extract (10% *w*/*v* in sterile water), sterile water as a negative control, and MA as a positive control for 30 min at 37 °C in the presence of 5% CO_2_. Afterwards, the tissue was washed with PBS and incubated at 37 °C and 5% CO_2_ for 2 h. Subsequently, the tissue was incubated with the MTT solution (1 mg/mL) at 37 °C for 3 h. The fluid was then replaced with isopropanol and incubated at room temperature for a further 2 h. Two aliquots of each sample were transferred into a 96-well plate, and the absorbance at 570 nm was assessed as previously described. The values obtained for the untreated control cells were taken as 100% of cell viability and used as a reference to calculate the cell viability % of each sample. The experiments were performed in triplicate (n = 3), and results are reported as means ± SD.

### 2.15. Human Cell Line Activation Test (h-CLAT)

The in vitro skin sensitizing potential test was performed in accordance with the OECD 442E guideline for testing of chemicals [37] and to the 158 EURL–ECVAM protocol (European Union Reference Laboratory for alternatives to animal testing) [38]. The assay was conducted on the human monocytic leukemia cell line THP-1 (ATCC TIB-202) cultured with RPMI culture medium containing 10% FBS, 0.05 mM 2-mercaptoethanol, 100 U/mL penicillin, and 100 μg/mL streptomycin. Firstly, it was necessary to select the concentrations to be used for the h-CLAT assay. The P extract (10% *w*/*v* in sterile water) was diluted in isotonic solution and then cell culture medium to obtain eight stock solutions mixed 1:1 (*v*/*v*) with cell suspensions (final extract concentrations: 53.50 mg/mL, 26.75 mg/mL, 13.38 mg/mL, 6.69 mg/mL, 3.34 mg/mL, 1.67 mg/mL, 0.84 mg/mL, and 0.42 mg/mL) and then placed into a 96-well plate (1.6 × 105 cells/well). Analogously, cells were treated with DNCB as a positive control and lactic acid as a negative control. After 24 h of incubation, cells were collected, re-suspended in FACS buffer containing propidium iodide, and subjected to cytometry analysis to determine the concentration that caused 25% of cell mortality, which will correspond to the highest concentration employable in the h-CLAT test. The latter was performed by treating THP-1 cells with P extract (tested concentrations: 24.08 mg/mL, 20.06 mg/mL, 16.72 mg/mL, 13.94 mg/mL, 11.61 mg/mL, 9.68 mg/mL, 8.07 mg/mL, and 6.72 mg/mL), cell culture medium as a negative control, and DNCB (4 μg/mL) as a positive control for 24 h at 37 °C in the presence of 5% CO_2_. Afterwards, cells were collected, re-suspended in the blocking solution (FACS buffer containing 0.01% of human gamma-globulins), incubated for 15 min at 4 °C and then centrifuged and incubated with fluorescent anti-CD86, anti-CD54, or mouse IgG1 (control isotype) antibodies at 4 °C for 30 min. Subsequently, cells were washed up and then re-suspended with FACS buffer containing propidium iodide to determine the expression levels of CD86 and CD54. Similarly, both treated (P extract and DNCB) and control (culture medium) cells were subjected to cytometry to evaluate the intrinsic fluorescence. CD86 and CD54 expressions were then calculated by measuring the fluorescence intensity and normalizing it to that of the control and to the basal one as follows:

$$RFI\;\%=\frac{{MFI}_{treated\;cells}-{\;MFI}_{treated\;cells\;with\;control\;isotype}\;}{{MFI}_{control\;cells}\;-\;{MFI}_{control\;cells\;with\;control\;isotype}}\;\times 100$$

where RFI is the relative fluorescence intensity and MFI is the mean fluorescence intensity for each experiment. The experiments were performed three times in triplicate (n = 9), and results are reported as means ± SD.

### 2.16. Development of the P Extract Loaded In Situ Gelling Buccal Formulation

The carefully weighted amount of each component was added in the required amount of citrate buffer pH 5.5 under magnetic stirring in the following order: ascorbic acid, potassium sorbate, xylitol, sodium metabisulfite, urea, PVP K30, P extract, benzyl alcohol, and sodium dehydrocholate. Each component was added after complete dissolution of the previously inserted one. Finally, the resulting solution was cooled at 4 °C, and then the carefully weighted amount of Pluronic F-127 was added to the cold solution and left under vigorous magnetic stirring in an ice bath for 3 h until a smooth, homogeneous, and fluid gel was obtained. The buccal formulation was prepared three times and stored at 4 °C in amber glass containers equipped with a spray dispenser.

### 2.17. pH Evaluation of the Buccal Formulation

The pH of the obtained buccal formulation was measured by using a pH meter HI 2211 pH/ORP Meter, Hanna Instrument (Woonsocket, RI, USA). The formulation was evaluated as such, without further water dilution. The measurement was repeated three times on each prepared formulation (n = 9), and results are reported as means ± SE.

### 2.18. In Vitro and Ex Vivo Temperature-Dependent Gelation

For the in vitro assessment of the temperature-dependent gelation, a watch glass was heated at 37 ± 1 °C, and then the formulation was sprayed on it. Then, some photographs were acquired to visually demonstrate the gelation of the starting fluid formulation. To highlight the temperature-dependent behavior, the same experiment was performed by spraying the formulation on an unheated watch glass. For the ex vivo experiment, a specimen of porcine buccal tissue was kept for 5 min in a beaker pre-filled with heated (37 ± 1 °C) simulated salivary fluid pH 6.8. The tissue specimen was then located into a Petri dish and tilted to better simulate the in vivo condition, and then the formulation was sprayed on it. A video was acquired to visualize what should happen when administering the proposed formulation as a buccal spray. The experiments were repeated by testing all three preparations.

### 2.19. Extensibility Profile of Buccal Formulation

The extensibility of a semi-solid dosage form is defined as the area occupied by a given amount of formulation subjected to a standard pressure between two glass plates performed at a defined temperature [39]. To determine the extensibility (or spreadability) of the thermosensitive buccal formulation at room temperature (25 ± 1 °C), 30 μL of the P extract-loaded buccal gel was put on the surface of a glass Petri dish, forming a drop with a previously marked 0.7 cm^2^ contact area with the surface. The extensibility of the formulation was evaluated by placing on the drop simply a second glass (5 g of weight) or a second glass plus a series of increasing weights (5, 10, 20, 50, and 100 g) for 60 s. For each weight, the diameter (cm) of the circle spread was measured and recorded to calculate the final surface area of the formulation as a function of the increasing weight applied, indicated as extensibility (cm^2^). To confirm the temperature-dependent gelation behavior of the formulation, the experiments were repeated at 37 ± 1 °C. In this case, the Petri dish and the second glass were pre-heated in an oven, and then the experiments were carried out on a heating plate. The formulation was put on the hot Petri dish and kept for 1 min before starting the experiment. Each experiment was performed in triplicate, and results are reported as means (n = 3) ± SE.

### 2.20. Evaluation of the Antioxidant Power of the Buccal Formulation by DPPH Assay

The DPPH assay was performed as reported above, by adding 100 µL of a 100 mg/mL methanolic solution of the formulation into a quartz cuvette containing 2 mL of DPPH stock solution (40 µg/mL in methanol). The resulting solutions were well-mixed and immediately subjected to UV–Vis measurements every 5 min for 1 h at room temperature. Blank control samples were prepared analogously by adding 100 µL of methanol. To evaluate the additive antioxidant behavior of the P extract and the other antioxidants inserted as preservatives, the experiments were also performed using a formulation prepared without potassium sorbate, sodium metabisulfite, or ascorbic acid. Each experiment was performed in triplicate on each prepared formulation (n = 9), and the results are expressed as the percentage amount of residual DPPH (means ± SE) as a function of time.

### 2.21. Ex Vivo Studies on Permeation/Penetration of Polyphenols Through Porcine Mucosal Tissues

#### 2.21.1. Tissue Preparation

The mucosae of the ventral surface of the tongue (sublingual) or from the vestibular area of the retromolar trigone (buccal) were collected from freshly slaughtered domestic, 6–8-month-old pigs intended for human consumption (ethical approval unrequired). The specimens were washed, cleaned of any excess tissue, and treated for 1 h with a trehalose-containing isotonic solution (5% *w*/*v*) before storage at −80 °C for at least one week. At the time of ex vivo experiments, tissue samples were defrosted, washed with PBS, and then subjected to thermal shock to remove the connective tissue and obtain thin layers of buccal and sublingual specimens, as reported in the literature [40,41].

#### 2.21.2. Ex Vivo Permeation and Penetration Assay

To evaluate polyphenol permeation through the porcine buccal and sublingual mucosae, vertical Franz-type diffusion cells (Permeagear, amber, unjacketed, flat flange joint, 11.28 mm orifice diameter, 8 mL acceptor volume, SES GmbH-Analysesysteme, Bechenheim, Germany) were used as a two-compartment open model. Firstly, the obtained mucosae were equilibrated in isotonic solution overnight, then the Franz cells were mounted using adequate sections of the mucosa as a membrane between the acceptor and the donor chambers, filled with citrate buffer (pH 5.5), and left to settle at 37.0 ± 0.5 °C for 15 min. Subsequently, the donor fluid was replaced with 0.4 mL of the proposed formulation, and every 5 min, 0.4 mL samples were withdrawn from the acceptor compartment and immediately replaced with fresh acceptor fluid to maintain the sink conditions. Samples were immediately frozen, freeze-dried, and then redispersed in 0.4 mL of methanol, centrifuged, and the clear supernatant was subjected to HPLC-DAD analyses (as described above) to quantify the permeated amount of GA, RSV, and QRC, followed as reference polyphenols of the P extract. Each experiment was carried out at 37.0 ± 0.5 °C under continuous magnetic stirring in the dark and repeated 6 times for each time point (15 min, 30 min, and 3 h). At the end of the permeation experiments, the Franz cells were disassembled, and the porcine mucosae were washed (2 mL of distilled water) and then subjected to methanolic extraction of the polyphenols eventually entrapped. The extraction was carried out by dipping the tissue for 2 min in 2 mL of warm methanol (55 ± 5 °C) and repeating the procedure twice. The extraction liquors were collected into a 5 mL flask and brought to volume with methanol. The amount of GA, RSV, and QRC was determined by HPLC-DAD analyses as described above. Results are reported as means (n = 6) ± SE.

### 2.22. Data Analysis

The data are expressed as mean ± standard error (SE) or standard deviation (SD). All differences were statistically evaluated with Student’s *t*-test or the one-way analysis of variance (ANOVA or F-test) with the minimum levels of significance with *p* < 0.05.

## 3. Results and Discussion

### 3.1. BB Recovery and Green Extraction by Maceration

The recovery of polyphenols from waste BB by green extraction recently emerged as a virtuous approach to the current sustainability point of view. To enhance the “green soul” of the basic waste re-evaluation purpose, several other aspects need to be carefully evaluated, e.g., the cost, the scalability, and the environmental impact of the chosen extraction procedure. These aspects have been previously considered by proposing a cost-effective and easily scalable extraction procedure by simple maceration as well as by employing unconventional extraction solvents among the liquid hydrophilic raw material that is presently well-known and used in the pharmaceutical and cosmetic fields. Indeed, our previous research investigated the effect of the unconventional extraction solvent on the composition and antioxidant properties of the resulting extracts [27]. This previous work evaluated 5 different pure ‘solvents’: PEG200, PEG400, PEG600, propylene glycol, and glycerine. Among them, PEG200 and propylene glycol gave the best results and were then selected to perform the further here-reported studies. The first scope of the present work was thus to confirm the previously obtained data with PEG200 (P) and propylene glycol (G) by recovering polyphenols from another lot of waste BB (to ensure the reproducibility of the proposed waste recycle) and to further evaluate the success of the extraction procedure when using mixtures of the two mentioned liquid excipients instead of the stand-alone ones. As reported in Table 1 (see Materials and Methods Section) the extraction procedure by maceration was performed by using PEG200, propylene glycol, and their 7:3, 5:5, and 3:7 (*w*/*w*) mixtures. To confirm the previous data and further compare the novel results, the maceration process was carried out as follows: (i) in the dark, to avoid polyphenol photodegradation; (ii) at 25 °C, as it was previously observed that higher temperatures did not enhance the polyphenol recovery while could compromise their stability [42]; (iii) for 1 h under vigorous magnetic stirring, as shorter times were not sufficient to achieve a good extraction while longer times did not lead to an improvement. Immediately after maceration, the colored extracts were recovered by centrifugation and filtration and evaluated in terms of solvent loss % (as the waste BB swells once in contact with the solvent/mixture and creates a sort of gel, thus leading to liquid loss), density, and pH after water dilution. As reported in Table 3, the variation in solvent/mixture used did not significantly affect the solvent loss %, which resulted in ≈30% (*w*/*w*). The density of each extract resulted in being quite close to that of the native solvent/mixture (ρ_P_ = 1.115 ± 0.005 g/mL; ρ_G_ = 1.038 ± 0.004 g/mL), while the pH of the resulting water solutions was always acidic (≈4, due to the presence of acidic extracted substances such as polyphenols and must’s organic acids) with respect to the ≈6.5 pH values registered when diluting the same amount of PEG200, propylene glycol, or their mixtures with pure water.

### 3.2. Extracts Composition: Total Phenolic and Protein Contents and Evaluation of the Polyphenolic and Organic Acid Footprints by HPLC-DAD and HPLC-MS Analyses

First of all, the composition and total amount of polyphenols were evaluated, as they are the main biocompounds aimed at being recovered. The 3D plot chromatograms obtained by the HPLC-DAD analyses showed a complex composition and led to the identification and quantification of three molecules: gallic acid (GA), resveratrol (RSV), and quercetin (QRC). The whole phenolic content was then assessed by the well-known colorimetric Folin–Ciocalteu assay. The found data are reported in Table 4 and highlight a general reduction trend going from the P extract to the G one, probably due to enhanced solubility of the three selected molecules in PEG200 instead of propylene glycol. These results also confirmed the previously obtained data, thus verifying the reproducibility of polyphenol extraction from the waste BB.

As the 3D plots depicted the presence of several compounds, the extracts were further subjected to HPLC-MS/MS analyses aimed at both increasing the knowledge on the complex polyphenolic pool extracted and also evaluating the presence of organic acids coming from the must.

LC-MS/MS experiments allowed the identification of the polyphenolic profile of the extracts, detecting the presence of different classes of polyphenols (e.g., phenolic acids, flavonoids, and stilbenes). Qualitative and quantitative MS experiments confirmed the findings from HPLC-DAD data concerning the content of GA, RSV, and QRC, or, generally, the extraction trend (higher concentration in the P extract, which decreases until reaching the G one). Also, results from the Folin–Ciocalteu assay regarding the TPC for each extract were confirmed, highlighting the P extract as the best one. The latter emerged as the richest in polyphenols, as the total content was higher than in all the other extracts. Specifically, the total polyphenol content in the P extract is approximately double that in the G one, while intermediate amounts were found in the extracts obtained from different mixtures of PEG200 and propylene glycol. The quantitative data for individual polyphenols are reported in Table 5. As noticeable, G is not able to extract some of the evaluated polyphenols (i.e., gentisic acid, syringic acid, and apigenin-7-glu), which were founded only when the extraction solvent/mixture comprised PEG200, probably due to solubility issues.

Moreover, the organic acids generally present in the must were founded and quantified, as reported in Table 6 [43,44].

As a result, high amounts of tartaric and malic acids were found in all the extracts while a low content of lactic and citric acids was observed. Particularly, lactic acid content was directly proportional to the used amount of G as extraction solvent, thus giving the highest amount when using G as pure solvent. G was also greater than P to extract malic acid, as its content in the G extract was almost 2-fold higher than in the P one. However, the mixtures of P and G together resulted in the best extraction solvents for both tartaric and malic acids. Conversely, the amount of citric acid was the lowest in each sample, and the results obtained for the 5 extracts were superimposable. Overall, these results were in agreement with the previously reported acidic pH results for each extract. A key point to be considered is that the P extract generally possesses the highest phenolic content but also the lowest organic acid content. This result highlighted the enhanced selectivity of PEG200 for the purpose of the study, which is related to polyphenol recovery, while organic acid extraction is just a concomitant extraction. In any case, it should be pointed out that the acids coming from must are naturally occurring molecules that belong to the excipients generally included in cosmetics to act as buffers or moisturizing agents to cite some examples [45,46].

Additionally, as the scope of the fining process for which the BB is used is to minimize protein haze in the final product, the amount of protein extracted by the proposed unconventional solvents was determined by the colorimetric Bradford assay. The Total Protein Content (TPtC) was expressed in terms of mg equivalent of BSA as reported in Table 7. As observable, the P extract possesses the highest protein content, and the G extract exhibited the lowest one; the three P and G mixtures were almost superimposable and resulted in an intermediate behavior. It is relevant to emphasize that the presence of unknown proteins does not affect the cosmeceutical value of the proposed extracts. Actually, the here-founded proteins come from must filtration and are thus naturally occurring compounds in grape berries. They are removed from white musts solely to improve the characteristics of the final product in terms of stability over time and not because they pose a health risk [47]. Moreover, it is worth emphasizing that the TPtC values are about one order of magnitude lower than the TPC ones: these results suggest the extractive selectivity of the chosen solvents for polyphenols rather than proteins.

### 3.3. Comparison of Extracts’ Antioxidant Power by DPPH Assay

**Figure 1:**
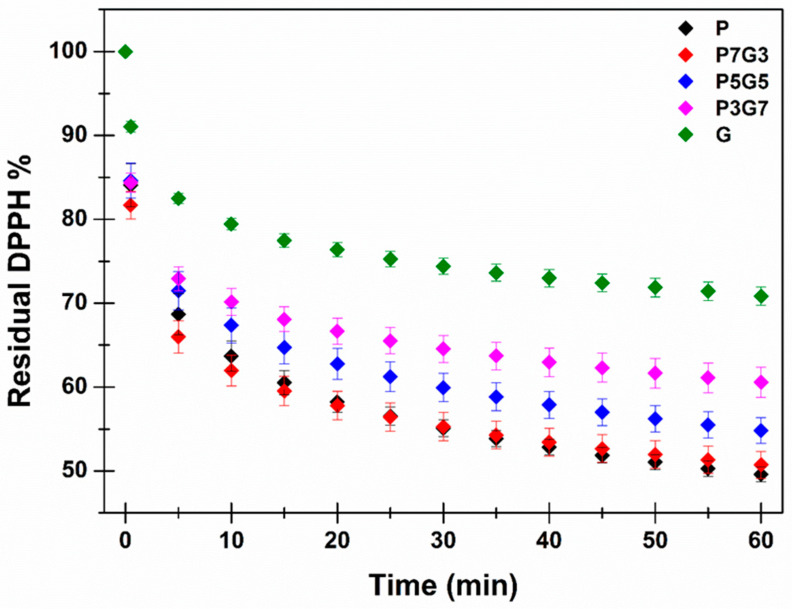
Antioxidant power of the green extracts reported as residual DPPH % over time until 1 h. Means (n = 18) ± SE (*p* < 0.05).

To the aim of producing a high value-added secondary raw material useful for cosmeceutical purposes, the scavenger properties of the prepared extracts were investigated by the DPPH assay, according to the already reported method. The obtained results are presented in Figure 1 as a percentage of residual DPPH as a function of time and processed in terms of antioxidant power expressed as gallic acid equivalents (GAE) mg per gram of extract calculated at different time points, as reported in Table 8. As the extracts contain a complex pool of molecules, each of which is able to react differently with the DPPH over time, the GAE values were calculated at 3 different incubation times: 10, 30, and 60 min. Indeed, GA is generally used as a reference molecule, but, as a pure molecule, it possesses a proper kinetic of reaction. When constructing the GA standard curves of reaction with the DPPH, they sharply decrease in the first 10 min while depicting a plateau from almost 10 min to the end of the experiment [48]. In contrast, as reported in Figure 1, the extracts have a complex kinetic reaction with the DPPH, leading to a continuous decreasing trend resulting in no plateau reaching also after 1 h of incubation. The consequence of these different behaviors results in the growing GAE values observed over time and reported in Table 8. As observable, the data collected by the DPPH assay are fully in agreement with the HPLC-DAD, TPC, and HPLC-MS results: the highest phenolic content confers to the P extract the greatest antioxidant activity. Minor differences were highlighted between the P and the P7G3 extracts: apparently the high amount of PEG200 in the P7G3 extract is sufficient to maximize the extraction process, giving results quite close to the maximum achievable ones in the employed experimental condition.

Based on the here-reported characterization, the P extract emerged as the better one, as it possesses the highest phenolic content and consequently antioxidant power. Even if the P7G3 extract is quite close to the P one, especially in terms of scavenger properties, the use of a single pure solvent could be preferable to ensure reproducibility as well as to propose a simple process for industrialization. Indeed, the here-reported investigations highlight that PEG200 is certainly a better extraction solvent for the polyphenols entrapped into the waste BB than propylene glycol. Consequently, when the mixture mainly consists of PEG200, the extraction procedure gave better results, while a reduction trend is always observable when increasing the propylene glycol content. The previous preparation of a solvent mixture could determine a source of error, leading to less reproducible results. Thus, the use of PEG200 as such is clearly preferable. Accordingly, further investigations were conducted only on the P extract.

### 3.4. In Vitro Validation of the P Extract as a Novel Cosmetic Raw Material

A valuable cosmeceutical excipient should be stable over time. The stability of the P extract was then monitored monthly for 1 year by repeating the previously mentioned assays. In Table 9 the obtained data for the 6- and 12-month time points are reported and compared to the starting data of the freshly prepared P extract. As noticeable, the amount of RSV and QRC decreased by 10 and 6%, respectively, after one year of storage, while GA concentration grew by more than 6% after one year; the latter could be attributable to some modification occurring in the polyphenols pool, thus releasing GA molecules from more complex structures. However, these changes did not significantly affect the whole extract characteristics, as the TPC and TPtC values remained almost constant. Finally, the antioxidant power of the extract seemed to increase over time. This could again be attributable to some variation in terms of the complex pool of polyphenols leading to finally obtaining molecules characterized by a different kinetic and power of reaction with the DPPH free radical with respect to the starting ones. To summarize, the P extract can be considered stable over time.

**Figure 2:**
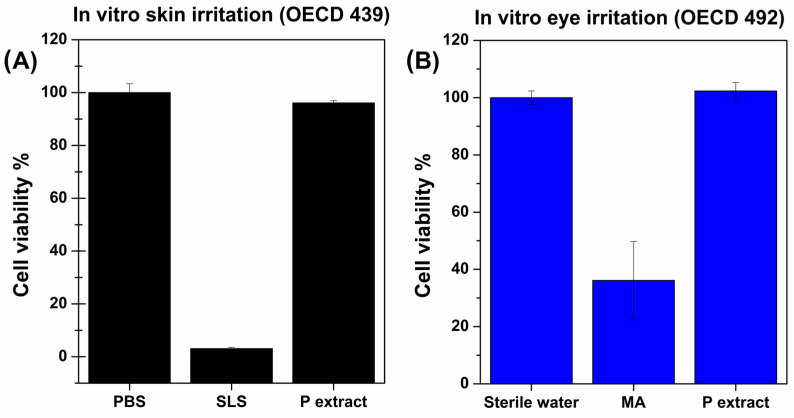
In vitro (**A**) skin and (**B**) eye irritation assays: cell viability % referred to control cells (treated with PBS or sterile water) after treatment with the positive control (SLS or MA) and the P extract (10% *w*/*v* solution in sterile water). The experiments were conducted in accordance with the OECD 439 and 492 guidelines by using a 3D human artificial skin model (EpiDerm^TM^) or a 3D human corneal epithelium model (EpiOcular^TM^), respectively. Means (n = 3) ± SD (*p* < 0.05).

In order to propose this novel polyphenol-enriched excipient for cosmeceutical purposes, the assessment of its safety is of crucial importance. To this aim, the biological characterization was carried out according to the Organisation for Economic Cooperation and Development (OECD) Guidelines for the Testing of Chemicals. The latter are a unique tool for determining the potential effects of chemicals on human health. Accepted internationally as standard methods for safety testing, the guidelines are used by professionals in industry, academia, and government involved in the testing and assessment of chemicals (industrial chemicals, pesticides, personal care products, etc.). These guidelines are continuously expanded and updated to ensure they reflect the state of the art and meet the regulatory needs. The first biological evaluations required to develop and register a novel cosmetic raw material are related to its skin and eye irritation potential (respectively OECD 439 and 492) as well as its skin sensitizing power (OECD 442E). As reported in Figure 2, the P extract did not depict any skin or eye irritation as the cell viability percentage values are quite close to the control ones, while the used positive controls determined unequivocal cell toxicity.

**Figure 3:**
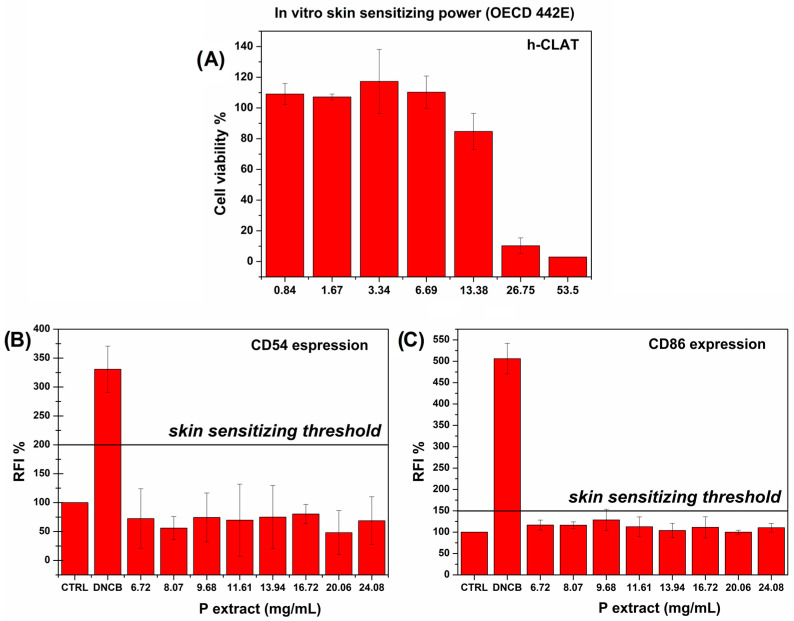
In vitro skin sensitizing power against the THP-1 cell line (ATCC TIB-202): (**A**), preliminary studies of cell viability % referred to control cells to evaluate the maximum P extract dose useful to evaluate the (**B**) CD54 and (**C**) CD86 expression by fluorescence. Data are compared to a negative control treated with cell culture medium and to a positive control treated with DNCB. The skin sensitizing threshold according to the OECD 442E is highlighted. Means (n = 9) ± SD (*p* < 0.05).

To assess the sensitizing power of the P extract, a composite in vitro test was performed in accordance with the OECD 442E. Firstly, the cytocompatibility range of concentration to be used for the further real test aimed at evaluating the skin sensitizing power was determined (Figure 3A). The sensitizing power was then assessed by evaluating the level of CD54 and CD86, which are known co-stimulatory molecules expressed during the process of skin sensitization due to immune response caused by antigen presentation [49]. In the graphs in Figure 3B,C, the threshold levels for CD54 and CD86, respectively, are highlighted.

As observable, the P extract did not enhance the production of both markers, which quantities remain similar to those of the untreated control cells and, in any case, below the threshold values. To summarize, the P extract possesses all the required characteristics to be a powerful, high-value-added excipient of cosmeceutical interest.

### 3.5. Development and Characterization of the P Extract Loaded Oral Spray: Temperature-Dependent Behavior and Antioxidant Power Evaluation

Once validated, the P extract was directly inserted into a liquid formulation intended for daily oral care. The aim of the formulation process was to obtain an easily administrable and patient-friendly cosmeceutical suitable to simply be included in a daily routine. To achieve this goal, a fluid formulation was prepared according to the complete *w*/*w* percentage composition reported in Table 10.

However, as the main drawback of liquid oral formulations is the extremely poor retention time on the mucosal surface, Pluronic F-127 was chosen as the gelling polymer due to its temperature-dependent gelation behavior. Indeed, this polymer leads to extreme fluid dispersion at room temperature (~25 °C), while gels at body temperature [50]. This dual behavior could be of crucial importance, as the starting fluidity of the formulation at room temperature will allow for ease of administration and sprayability, while the formation of a viscous gel once in contact with the oral mucosae will enhance retention time on them, allowing polyphenols to distribute on the tissues and accumulate therein. The formulation was loaded with high amounts of the P extract (30% *w*/*w*), also including xylitol as a sweetener and some preservatives (both antimicrobials and antioxidants), and was embellished by the addition of urea and sodium dehydrocholate, chosen as useful penetration enhancers for polyphenols [41]. The formulation was prepared by dissolving all components in citrate buffer pH 5.5 in order to preserve and stabilize polyphenols [51]. The resulting in situ-gelling buccal formulation was fluid, colored, and clear with a pH value of 5.35 ± 0.20.

**Figure 4:**
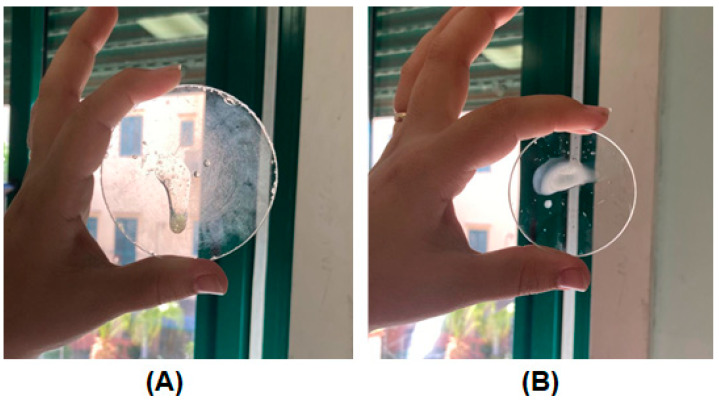
In vitro qualitative assay to verify the temperature-dependent gelation behavior of the buccal formulation when sprayed on a watch glass at (**A**) room temperature and (**B**) 37 ± 1 °C.

The temperature-dependent gelation behavior was qualitatively evaluated in vitro and ex vivo. For the in vitro assay, the formulation, kept at room temperature, was sprayed on two watch glasses: one kept at room temperature and the other pre-heated at 37 °C in an oven. The temperature-dependent comportment is clearly highlighted in Figure 4.

Furthermore, the same experiment was performed ex vivo by spraying the buccal formulation on the surface of a pre-heated porcine specimen. A video was recorded to visually highlight the immediate occurring interaction between the formulation and the buccal mucosa, leading to no droplet dripping even after applying several spray aliquots (see Supplementary Materials, Video S1).

**Figure 5:**
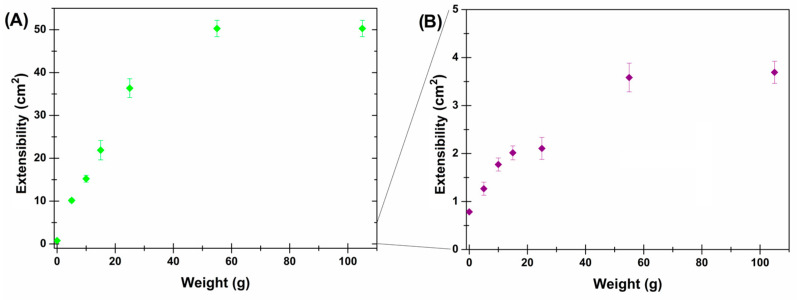
Extensibility profile measured by increasing applied weights of the thermosensitive formulation kept at (**A**) 25 ± 1 °C (green rhombus) and (**B**) 37 ± 1 °C (purple rhombus) (*p* < 0.01).

Once administered, the thermosensitive formulation should be distributed on the buccal mucosa and coat the epithelium, so a study of the extensibility of the formulation to evaluate the temperature-dependent behavior was performed. As reported in Figure 5, at room temperature, a drop of the fluid formulation spreads proportionally to the applied weight until reaching a plateau in which it occupies a surface up to 64 times the initial one. In contrast, at body temperature, the formulation gel, and a drop spreads until it occupies a surface up to less than 5 times the initial one. In the latter case, a bimodal curve is observable as the plateau seemed to be reached by adding weights of 25 g. Nevertheless, a significant weight increase (from 25 to 55 g) resulted in a doubled extensibility, which, although it remained constant even for high applied weights (up to 105 g). This thermosensitive extensibility behavior is adequate for the oromucosal formulation objectives: a liquid that can be easily sprayed from its device and able to cover the mucosa without slipping away, thus avoiding the risk of loss due to swallowing.

The antioxidant power of the formulation as a whole was then evaluated by the DPPH assay according to the methodology previously described to test the extracts. The obtained results highlighted the maintenance of the antioxidant trend observed for the P extract. Again, for the buccal gel, the scavenger activity grew over time. GAE values after 10, 30, and 60 min of incubation resulted in 0.483 ± 0.003 mg/g, 0.541 ± 0.003 mg/g, and 0.574 ± 0.003 mg/g, respectively. However, as the formulation contains antioxidants inserted as preservatives to protect polyphenols, they can certainly affect the DPPH results. To define the actual scavenging properties of the P extract when loaded into the proposed buccal gel, the formulation was then prepared in the absence of potassium sorbate, sodium metabisulfite, and ascorbic acid. The latter preservatives-free gel was subjected to the DPPH assay, resulting in growing GAE values over time and particularly equal to 0.290 ± 0.005 mg/g, 0.341 ± 0.002 mg/g, and 0.373 ± 0.005 mg/g after 10, 30, and 60 min, respectively (statistical analyses by comparing the GAE values in the presence or absence of preservatives gave *p* < 0.01). As observable, the final antioxidant properties of the complete formulation are the sum of the P extract and preservatives contributions. However, it is worth noticing that about 60% of the GAE value at each time point considered is attributable to the P extract.

### 3.6. Evaluation of Polyphenol Entrapment into the Oral Mucosae by Ex Vivo Assays Following Oral Spray Administration

**Figure 6:**
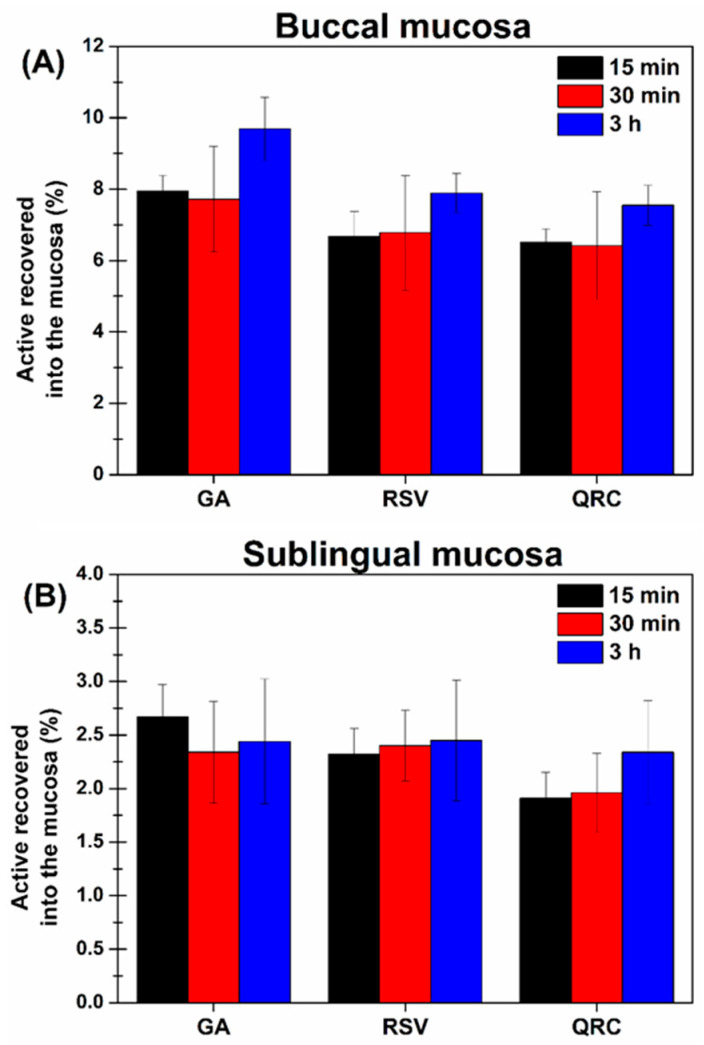
Percentage amount of GA, RSV, and QRC entrapped into the (**A**) buccal and (**B**) sublingual mucosae after different incubation times with the buccal in situ gelling formulation. Means (n = 6) ± SE (*p* < 0.05).

Finally, ex vivo tests were carried out to evaluate the ability of the polyphenols contained in the in situ gelling formulation to penetrate and accumulate in the oral mucosae. The experiments were performed by using both the buccal and the sublingual mucosae, as the proposed formulation is generally intended for the daily care of the whole oral cavity. Polyphenols’ permeation and penetration were studied following the GA, RSV, and QRC behavior. Particularly, it was observed that the mentioned reference polyphenols were not able to permeate through the mucosae and reach the acceptor chamber even if the three of them are characterized by different physico-chemical properties (e.g., logP). Moreover, as samples were subjected to HPLC-DAD analyses, it was possible to notice that no peaks were detectable until 3 h in the wavelength range 190–640 nm. These are positive results, as cosmetic/cosmeceutical formulations should not permit actives to reach the systemic circulation but must let them act locally. However, the topical activity is clearly strictly associated with polyphenol accumulation into the target tissues. Consequently, the amount of GA, RSV, and QRC entrapped into the buccal and sublingual tissues after different incubation times was determined. As depicted in Figure 6, the percentage amount of the three considered biomolecules is higher in the buccal mucosa than the sublingual one. This is probably due to the hydrophobicity of the buccal tissue, leading to better interaction with the lipophilic polyphenols. This feature should be less relevant for GA, which is water-soluble; however, it should also be considered that the buccal mucosa is thicker than the sublingual one (250 ± 25 μm vs. 80 ± 8 μm), therefore having a greater accumulation volume by the same contact surface [41,52]. It is worth noticing that, for all three actives, both in the buccal and sublingual mucosae tests, no significant variation in terms of percentage of polyphenols recovered into the tissue was observable over time. These results suggest a rapid interaction between the actives and the target tissue, resulting in their accumulation also after short contact times. Additionally, the weak increase in accumulation over time could suggest that a saturation phenomenon of the membrane caused by the experimental setup has been established.

Considering that once in contact with the mucosae, the formulation should likely remain in situ for short periods of time despite its gelation, the data obtained after 15 min of incubation were further manipulated in order to determine the amount of each quantified polyphenol per unit surface area. As reported in Table 11, the latter values are in the nanogram range, but it is well known that polyphenols are effective even at very low concentrations [53], and thus the proposed formulation should be useful for the targeted well-being cosmeceutical purposes.

## 4. Conclusions

Through this work, the starting hypotheses were all confirmed, and the highlighted aims were all achieved. First, BB was confirmed as a sustainable source of polyphenols, which could be extracted by unconventional “solvents” in order to obtain a novel enriched raw material of cosmetic interest. Secondly, the ultimate aim of obtaining an effective cosmetic product that harnesses the potential of polyphenols was pursued with a continuous focus on green procedures, sustainability, and the circular economy. The selection and validation of PEG200 as the best extraction solvent for the polyphenols entrapped into the waste bentonite was a key innovation in this work, as it highlighted how choosing a liquid, hydrophilic raw material of cosmetic and pharmaceutical interest, instead of traditional extraction solvents, can be a winning strategy to obtain a cost-effective product. Indeed, the P extract could itself be directly marketable as a secondary raw material with high value added according to a waste-to-market approach. The P extract was thus validated as a novel cosmetic raw material through appropriate in vitro tests conducted according to OECD guidelines. Finally, it was effectively incorporated into a liquid formulation suitable for daily oral care and able to promote a great uptake of functional polyphenols into the oral mucosae even following short contact times.

## Figures and Tables

**Table 1:** Extraction solvents and mixtures used for BB maceration and sample formula codes.

Formula Code	Extraction Solvent/Mixture
P	PEG200
P7G3	PEG200: Propylene Glycol = 70:30 (*w*/*w*)
P5G5	PEG200: Propylene Glycol = 50:50 (*w*/*w*)
P3G7	PEG200: Propylene Glycol = 30:70 (*w*/*w*)
G	Propylene Glycol

**Table 2:** DPPH standard curves used to quantify the antioxidant power of the extracts as a function of time.

Time (min)	Residual DPPH %	R
10	90.65 − 1153.55 × [mg/mL]	0.996
30	91.38 − 1293.95 × [mg/mL]	0.999
60	90.65 − 1338.35 × [mg/mL]	0.999

**Table 3:** Characteristics of the green extracts in terms of solvent loss % (n = 6), density, and pH after water dilution (n = 18). Means ± SE (*p* < 0.05).

Formula Code	Solvent Loss %	Density	pH
P	30.73 ± 1.80	1.142 ± 0.005	4.30 ± 0.12
P7G3	30.76 ± 3.54	1.101 ± 0.003	4.23 ± 0.15
P5G5	34.06 ± 2.22	1.097 ± 0.003	4.23 ± 0.22
P3G7	27.16 ± 1.46	1.081 ± 0.009	4.05 ± 0.36
G	29.34 ± 1.48	1.040 ± 0.005	4.32 ± 0.19

**Table 4:** Polyphenols quantitative data for each extract: concentration (µg/mL) ± SE of GA, RSV, and QRC determined by HPLC-DAD analyses and TPC expressed as mg equivalent of GA per gram of extract ± SE obtained by the Folin–Ciocalteu assay. Means (n = 18) ± SE (*p* < 0.01).

Formula Code	HPLC-DAD Analyses			Folin–Ciocalteu Assay
	GA (µg/mL)	RSV (µg/mL)	QRC (µg/mL)	TPC (mg/g)
P	17.14 ± 0.80	2.12 ± 0.06	60.88 ± 2.28	3.14 ± 0.17
P7G3	17.58 ± 0.46	1.84 ± 0.05	42.81 ± 1.87	2.93 ± 0.14
P5G5	18.00 ± 0.71	1.75 ± 0.08	38.78 ± 2.61	2.70 ± 0.16
P3G7	16.54 ± 0.90	1.63 ± 0.11	31.98 ± 2.04	2.78 ± 0.11
G	15.11 ± 1.13	1.50 ± 0.06	25.85 ± 1.82	1.85 ± 0.10

**Table 5:** Quantitative evaluation of the polyphenolic footprint in each extract by LC-MS/MS analyses: concentration (µg/mL) of the founded biocompounds. Means (n = 18). SD was always lower than 5% (*p* < 0.05).

	P (µg/mL)	P7G3 (µg/mL)	P5G5 (µg/mL)	P3G7 (µg/mL)	G (µg/mL)
Gallic Acid	24.00	19.84	13.13	15.56	12.70
Gentisic Acid	7.34	15.14	5.83	5.65	ND
Catechin	50.37	82.11	47.85	57.21	51.68
Caffeic Acid	18.96	25.63	15.59	17.53	18.04
Syringic Acid	9.30	6.59	4.30	4.03	ND
Epicatechin	25.00	25.44	16.43	19.60	22.90
Trans-OH Cinnamic	39.90	36.80	27.70	31.63	34.10
Rutin	2.90	2.54	2.32	2.32	2.35
Resveratrol	1.50	1.66	1.05	1.28	1.60
Apigenin_7_glu	0.90	0.59	0.58	0.58	ND
Kaempferol	31.00	31.17	20.58	19.87	14.40
Quercetin	330.30	260.62	120.19	123.95	156.50
⅀ Polyphenols	541.47	508.13	275.55	299.21	314.27

ND: not detected.

**Table 6:** Quantitative determination of the organic acids in each extract by LC-MS/MS analyses: concentration (µg/mL) of the founded biocompounds. Means (n = 18). SD was always lower than 5% (*p* < 0.05).

Formula Code	Lactic Acid (µg/mL)	Tartaric Acid (µg/mL)	Citric Acid (µg/mL)	Malic Acid (µg/mL)
P	13.1	1459.5	3.8	833.9
P7G3	28.8	2026.8	4.1	1607.3
P5G5	32.8	2068.5	3.7	1748.5
P3G7	33.9	2476.3	4.4	1620.0
G	57.4	1864.0	3.9	1511.1

**Table 7:** Bradford assay: TPtC expressed as mg equivalent of BSA per gram of each extract (n = 18) ± SE (*p* < 0.05).

Formula Code	TPtC (mg/g)
P	0.252 ± 0.013
P7G3	0.198 ± 0.020
P5G5	0.198 ± 0.016
P3G7	0.210 ± 0.009
G	0.166 ± 0.016

**Table 8:** DPPH assay: quantification of the antioxidant power of each extract after 10, 30, and 60 min of incubation. Data are expressed as gallic acid equivalent (GAE) mg per gram of each extract (n = 18) ± SE (*p* < 0.05).

Formula Code	GAE (mg/g)		
	10 min	30 min	60 min
P	1.072 ± 0.081	1.339 ± 0.035	1.579 ± 0.028
P7G3	1.234 ± 0.078	1.385 ± 0.065	1.479 ± 0.060
P5G5	0.992 ± 0.086	1.195 ± 0.060	1.317 ± 0.053
P3G7	0.867 ± 0.069	1.012 ± 0.063	1.097 ± 0.068
G	0.483 ± 0.031	0.652 ± 0.043	0.763 ± 0.046

**Table 9:** Stability studies of the P extract stored in the dark at 4 °C for a total of 12 months: concentration (µg/mL) ± SE of GA, RSV, and QRC determined by HPLC-DAD analyses; TPC expressed as mg equivalent of GA per gram of extract ± SE; TPtC expressed as mg equivalent of BSA per gram of extract ± SE; antioxidant power expressed as GAE mg per gram of extract ± SE. Means ± SE (n = 12) of the data collected after 6 and 12 months of storage (*p* < 0.05).

Performed Assay	Parameter	P_t=0_	P_t=6_	P_t= 2_
HPLC-DAD	GA (μg/mL)	17.14 ± 0.80	17.25 ± 1.14	18.27 ± 0.57
	RSV (μg/mL)	2.12 ± 0.06	1.72 ± 0.10	1.92 ± 0.42
	QRC (μg/mL)	60.88 ± 2.28	52.61 ± 3.88	57.30 ± 3.81
Folin–Ciocalteu	TPC (mg/g)	3.14 ± 0.17	3.20 ± 0.10	3.18 ± 0.12
Bradford	TPtC (mg/g)	0.25 ± 0.01	0.23 ± 0.01	0.23 ± 0.01
DPPH	GAE_60min_ (mg/g)	1.58 ± 0.03	1.79 ± 0.02	1.83 ± 0.06

**Table 10:** Percentage *w*/*w* composition of the in situ gelling buccal formulation loaded with the P extract.

Components	*w*/*w* %
P extract	30
Pluronic F-127	10
PVP K30	5
Xylitol	3
Benzyl alcohol	0.5
Potassium sorbate	0.5
Sodium metabisulfite	0.2
Ascorbic acid	0.05
Urea	0.05
Sodium dehydrocholate	0.05
Citrate buffer pH 5.5	50.65

**Table 11:** Amount of GA, RSV, and QRC (ng) accumulated into the buccal and sublingual mucosae per surface area when considering a contact time equal to 15 min. Means (n = 6) ± SE (*p* < 0.05).

	Buccal Mucosa	Sublingual Mucosa
	ng/cm^2^	ng/cm^2^
GA	163.77 ± 5.23	55.00 ± 3.60
RSV	16.69 ± 1.02	5.72 ± 0.42
QRC	475.88 ± 16.16	139.62 ± 10.21

## Data Availability

Data is contained within the article and Supplementary Materials.

## Supplementary Materials

The following supporting information can be downloaded at https://www.mdpi.com/article/10.3390/pharmaceutics16121612/s1, Table S1: LC-MS/MS parameter and LOD values for polyphenols identification and quantification in the 5 extracts; Table S2: LC-MS/MS parameters for organic acids identification and quantification in the 5 extracts; Video S1: Ex vivo temperature-dependent gelation studies.

## References

[1] Grace R. (2002). Cosmeceuticals: Functional Food for the Skin. Nat. Foods Merch..

[2] Mago Y., Sharma Y., Thakran Y., Mishra A., Tewari S., Kataria N. (2023). Next-Generation Organic Beauty Products Obtained from Algal Secondary Metabolites: A Sustainable Development in Cosmeceutical Industries. Mol. Biotechnol..

[3] Pandey A., Gurpoonam K.J., Sidharth S. (2023). Cosmeceuticals.

[4] He X., Wan F., Su W., Xie W. (2023). Research Progress on Skin Aging and Active Ingredients. Molecules.

[5] Yoshino F., Yoshida A., Nakajima A., Wada-Takahashi S., Takahashi S., Lee M.C. (2013). Alteration of the Redox State with Reactive Oxygen Species for 5-Fluorouracil-Induced Oral Mucositis in Hamsters. PLoS ONE.

[6] Rekha V.R., Sunil S., Rathy R. (2017). Evaluation of Oxidative Stress Markers in Oral Lichen Planus. J. Oral Maxillofac. Pathol..

[7] Csekes E., Račková L. (2021). Skin Aging, Cellular Senescence and Natural Polyphenols. Int. J. Mol. Sci..

[8] Arbeláez L.F.G., Pardo A.C., Fantinelli J.C., Schinella G.R., Mosca S.M., Ríos J.-L. (2018). Cardioprotection and Natural Polyphenols: An Update of Clinical and Experimental Studies. Food Funct..

[9] Angellotti G., Di Prima G., Belfiore E., Campisi G., De Caro V. (2023). Chemopreventive and Anticancer Role of Resveratrol against Oral Squamous Cell Carcinoma. Pharmaceutics.

[10] Rebas E., Rzajew J., Radzik T., Zylinska L. (2020). Neuroprotective Polyphenols: A Modulatory Action on Neurotransmitter Pathways. Curr. Neuropharmacol..

[11] Belfiore E., Di Prima G., Angellotti G., Panzarella V., De Caro V. (2024). Plant-Derived Polyphenols to Prevent and Treat Oral Mucositis Induced by Chemo-and Radiotherapy in Head and Neck Cancers Management. Cancers.

[12] Rana A., Samtiya M., Dhewa T., Mishra V., Aluko R.E. (2022). Health Benefits of Polyphenols: A Concise Review. J. Food Biochem..

[13] Piyaratne P.S., Leblanc R., Myracle A.D., Cole B.J.W., Fort R.C. (2022). Extraction and Purification of (E)-Resveratrol from the Bark of Conifer Species. Processes.

[14] Rogachev A.D., Salakhutdinov N.F. (2015). Chemical Composition of *Pinus sibirica* (Pinaceae). Chem. Biodivers..

[15] Pogorzelska-Nowicka E., Hanula M., Pogorzelski G. (2024). Extraction of Polyphenols and Essential Oils from Herbs with Green Extraction Methods–An Insightful Review. Food Chem..

[16] Aktaş H., Kurek M.A. (2024). Deep Eutectic Solvents for the Extraction of Polyphenols from Food Plants. Food Chem..

[17] Li C., Chen L., McClements D.J., Peng X., Xu Z., Meng M., Ji H., Qiu C., Long J., Jin Z. (2023). Encapsulation of Polyphenols in Protein-Based Nanoparticles: Preparation, Properties, and Applications. Crit. Rev. Food Sci. Nutr..

[18] Van Tran V., Moon J.-Y., Lee Y.-C. (2019). Liposomes for Delivery of Antioxidants in Cosmeceuticals: Challenges and Development Strategies. J. Control. Release.

[19] Chang Y., Shi X., He F., Wu T., Jiang L., Normakhamatov N., Sharipov A., Wang T., Wen M., Aisa H.A. (2022). Valorization of Food Processing Waste to Produce Valuable Polyphenolics. J. Agric. Food Chem..

[20] Tapia-Quirós P., Montenegro-Landívar M.F., Reig M., Vecino X., Cortina J.L., Saurina J., Granados M. (2022). Recovery of Polyphenols from Agri-Food by-Products: The Olive Oil and Winery Industries Cases. Foods.

[21] De Xavier Machado T.O., Portugal I.B.M., da Padilha C.V.S., Ferreira Padilha F., dos Santos Lima M. (2021). New Trends in the Use of Enzymes for the Recovery of Polyphenols in Grape Byproducts. J. Food Biochem..

[22] Xia E.Q., Deng G.F., Guo Y.J., Li H. (2010). Bin Biological Activities of Polyphenols from Grapes. Int. J. Mol. Sci..

[23] Hoss I., Rajha H.N., El Khoury R., Youssef S., Manca M.L., Manconi M., Louka N., Maroun R.G. (2021). Valorization of Wine-making By-products’ Extracts in Cosmetics. Cosmetics.

[24] Draghici-Popa A.M., Buliga D.I., Popa I., Tomas S.T., Stan R., Boscornea A.C. (2024). Cosmetic Products with Potential Photoprotective Effects Based on Natural Compounds Extracted from Waste of the Winemaking Industry. Molecules.

[25] Matos M.S., Romero-Díez R., Álvarez A., Bronze M.R., Rodríguez-Rojo S., Mato R.B., Cocero M.J., Matias A.A. (2019). Polyphenol-Rich Extracts Obtained from Winemakingwaste Streams as Natural Ingredients with Cosmeceutical Potential. Antioxidants.

[26] Morata A., Loira I. (2016). Grape and Wine Biotechnology.

[27] Di Prima G., Belfiore E., Angellotti G., De Caro V. (2024). Green Next-Generation Excipients Enriched in Polyphenols from Recovery of Grape Processing Waste Black Bentonite: Influence of Unconventional Extraction Solvents on Antioxidant Properties and Composition. Sustain. Chem. Pharm..

[28] Gal J.-Y., Fovet Y., Adib-Yadzi M. (2001). About a Synthetic Saliva for in Vitro Studies. Talanta.

[29] Di Prima G., Belfiore E., Migliore M., Scarpaci A.G., Angellotti G., Restivo I., Allegra M., Arizza V., De Caro V. (2022). Green Extraction of Polyphenols from Waste Bentonite to Produce Functional Antioxidant Excipients for Cosmetic and Pharmaceutical Purposes: A Waste-to-Market Approach. Antioxidants.

[30] Indelicato S., Houmanat K., Bongiorno D., Ejjilani A., Hssaini L., Razouk R., Charafi J., Ennahli S., Hanine H. (2023). Freeze Dried Pomegranate Juices of Moroccan Fruits: Main Representative Phenolic Compounds. J. Sci. Food Agric..

[31] (2021). Test No. 439: In Vitro Skin Irritation: Reconstructed Human Epidermis Test Method. OECD Guidelines for the Testing of Chemicals, Section 4.

[32] MatTek Corporation (2022). In Vitro EpiDerm^TM^ Skin Corrosion Test (EPI-200-SCT).

[33] (2024). Test No. 492: Reconstructed Human Cornea-like Epithelium (RhCE) Test Method for Identifying Chemicals Not Requiring Classification and Labelling for Eye Irritation or Serious Eye Damage. OECD Guidelines for Testing Chemicals.

[34] ECVAM (2015). DB-ALM DB-ALM Method Summary N° 164: EpiOcularTM Eye Irritation Test.

[35] MatTek Corporation (2021). EpiOcular^TM^ Eye Irritation Test (OCL-200-EIT).

[36] Alépée N., Leblanc V., Adriaens E., Grandidier M.H., Lelièvre D., Meloni M., Nardelli L., Roper C.S., Santirocco E., Toner F. (2016). Multi-Laboratory Validation of SkinEthic HCE Test Method for Testing Serious Eye Damage/Eye Irritation Using Liquid Chemicals. Toxicol. Vitr..

[37] (2024). Test No. 442E: In Vitro Skin Sensitisation. OECD Guidelines for the Testing of Chemicals, Section 4.

[38] ECVAM (2018). DB-ALM Protocol N° 158: Human Cell Line Activation Test (h-CLAT) Skin Sensitisation & Allergic Contact Dermatitis.

[39] Campaña-Seoane M., Peleteiro A., Laguna R., Otero-Espinar F.J. (2014). Bioadhesive Emulsions for Control Release of Progesterone Resistant to Vaginal Fluids Clearance. Int. J. Pharm..

[40] Angellotti G., Di Prima G., Scarpaci A.G., D’Agostino F., Campisi G., De Caro V. (2022). Spray-Dried Cytisine-Loaded Matrices: Development of Transbuccal Sustained-Release Tablets as a Promising Tool in Smoking Cessation Therapy. Pharmaceutics.

[41] Di Prima G., Angellotti G., Scarpaci A.G., Murgia D., D’agostino F., Campisi G., De Caro V. (2021). Improvement of Resveratrol Permeation through Sublingual Mucosa: Chemical Permeation Enhancers versus Spray Drying Technique to Obtain Fast-Disintegrating Sublingual Mini-Tablets. Pharmaceutics.

[42] Zhang H., Wang M., Xiao J. (2022). Stability of Polyphenols in Food Processing. Adv. Food Nutr. Res..

[43] Clarke R.J., Bakker J. (2004). Wine Flavour Chemistry.

[44] Todorov S.D., Alves V.F., Popov I., Weeks R., Pinto U.M., Petrov N., Ivanova I.V., Chikindas M.L. (2024). Antimicrobial Compounds in Wine. Probiotics Antimicrob. Proteins.

[45] Draelos Z.D. (2018). The Science behind Skin Care: Moisturizers. J. Cosmet. Dermatol..

[46] Liu J.-K. (2022). Natural Products in Cosmetics. Nat. Prod. Bioprospect..

[47] Sauvage F.-X., Bach B., Moutounet M., Vernhet A. (2010). Proteins in White Wines: Thermo-Sensitivity and Differential Adsorbtion by Bentonite. Food Chem..

[48] Di Prima G., Scurria A., Angellotti G., Belfiore E., Pagliaro M., Meneguzzo F., De Caro V., Ciriminna R. (2022). Grapefruit IntegroPectin Isolation via Spray Drying and via Freeze Drying: A Comparison. Sustain. Chem. Pharm..

[49] Yoshida Y., Sakaguchi H., Ito Y., Okuda M., Suzuki H. (2003). Evaluation of the Skin Sensitization Potential of Chemicals Using Expression of Co-Stimulatory Molecules, CD54 and CD86, on the Naive THP-1 Cell Line. Toxicol. Vitr..

[50] Grela K.P., Bagińska I., Burak J., Marciniak D.M., Karolewicz B. (2019). Natural Gums as Viscosity-Enhancers in Pluronic^®^ F-127 Thermogelling Solutions. Pharmazie.

[51] Angellotti G., Presentato A., Murgia D., Di Prima G., D’Agostino F., Scarpaci A.G., D’Oca M.C., Alduina R., Campisi G., De Caro V. (2021). Lipid Nanocarriers-Loaded Nanocomposite as a Suitable Platform to Release Antibacterial and Antioxidant Agents for Immediate Dental Implant Placement Restorative Treatment. Pharmaceutics.

[52] De Caro V., Angellotti G., D’Agostino F., Di Prima G. (2022). Buccal Thin Films as Potent Permeation Enhancers for Cytisine Transbuccal Delivery. Membranes.

[53] Ungvari Z., Bagi Z., Feher A., Recchia F.A., Sonntag W.E., Pearson K., De Cabo R., Csiszar A. (2010). Resveratrol Confers Endothelial Protection via Activation of the Antioxidant Transcription Factor Nrf2. Am. J. Physiol. Heart Circ. Physiol..

