# Supplementary material for: Characterization and Safety Assessment of a Novel Antioxidant Excipient from Sustainable Recovery of Grape Processing Waste Bentonite Designed to Develop a Thermosensitive Buccal Spray for Oral Cavity Wellness
Source: Pharmaceutics. 2024 Dec 19;16(12):1612. doi: 10.3390/pharmaceutics16121612 (PMC11678101; doi:10.3390/pharmaceutics16121612)
Supplement: Supplementary file 1 [file pharmaceutics-16-01612-s001.zip › Di Prima et al_supplementary materials.pdf]

pharmaceutics

MDPI

Supplementary Materials

Characterization and safety assessment of a novel antioxidant excipient from sustainable recovery of grape processing waste bentonite designed to develop a thermosensitive buccal spray for oral cavity wellness

Giulia Di Prima[1,*], Elena Belfiore[2], Cecilia La Mantia[1], Serena Indelicato[1], Giuseppe Avellone[1,3], Viviana De Caro[1,3]

[1]Dipartimento di Scienze e Tecnologie Biologiche, Chimiche e Farmaceutiche (STEBICEF), University of Palermo, Via Archirafi 32, 90123, Palermo, Italy. cecilialamantina000@gmail.com (CLM); serena.indelicato@unipa.it (SI); beppe.avellone@unipa.it (GA); viviana.decaro@unipa.it (VDC)

[2]Dipartimento di Medicina di Precisione in Area Medica, Chirurgica e Critica (Me.Pre.C.C.), University of Palermo, Via L. Giuffrè 5, 90127, Palermo, Italy. elena.belfiore@unipa.it (EB)

[3]Centro Interdipartimentale di Ricerca Riutilizzo Bio-Based Degli Scarti da Matrici Agroalimentari (RIVIVE), University of Palermo, 90128 Palermo, Italy

[*]Correspondance: Dr. Giulia Di Prima, giulia.diprima@unipa.it (GDP), +3909123896826

Data reported as supplementary material:

- **Table S1.** LC-MS/MS parameter and LOD values for polyphenols identification and quantification in the 5 extracts
- **Table S2.** LC-MS/MS parameters for organic acids identification and quantification in the 5 extracts
- **Video V1.** Ex vivo temperature-dependant gelation studies

Table S1. LC-MS/MS parameter and LOD values for polyphenols identification and quantification in the 5 extracts

	Precursor Ion (m/z) [M-H]⁻	Product Ion (m/z)	Collision Energy (V)	RF Lens (V)	LOD (µg/L)
Gallic Acid	169	79	24	101	30
	169	125	14	101	
Vanillic Acid	177	123	20	105	32
	177	152	20	105	
Ferulic Acid	193	134	15	99	22
	193	178	13	99	
Chlorogenic Acid	353	179	45	180	26
	353	191	45	180	
Catechin	289	203	20	147	28
	289	245	15	147	
Mandelic Acid	151	77	18	65	26
	151	107	10	65	
Gentisic Acid	153	108	22	90	24
	153	109	14	90	
Syringic Acid	197	153	12	100	23
	197	182	14	100	
Caffeic Acid	179	107	25	101	26
	179	135	16	103	
OH-Cynnamic	163	93	31	90	27
	163	119	14	90	
Rutin	609	271	60	299	26
	609	300	38	299	
Resveratrol	227	143	27	156	25
	227	185	20	156	
Apigenin-7Glu	433	269	20	123	24
	433	271	20	123	
Quercetin	301	151	18	166	25
	301	179	21	166	
Kaempferol	285	202	20	195	26
	285	239	29	195	
Cumaric Acid	163	93	31	91	27
	163	119	13	91	
Apigenin	269	117	35	178	25
	269	151	25	178	

Table S2. LC-MS/MS parameters for organic acids identification and quantification in the 5 extracts

	**Precursor Ion (m/z) [M-H]⁻**	**Product Ion (m/z)**	**Collision Energy (V)**	**RF Lens (V)**
Lactic acid	89	42.8	11	65
	89	44.8	12	165
Tartaric Acid	149	72.8	16	105
	149	86.8	13	105
Malic Acid	132.9	71	16	79
	132.9	114	19	79
Citric Acid	91	86.8	12	79
	91	110.8	17	79
